# Radix Rehmanniae and Corni Fructus against Diabetic Nephropathy via AGE-RAGE Signaling Pathway

**DOI:** 10.1155/2020/8358102

**Published:** 2020-12-02

**Authors:** Jing Chen, Yuping Chen, Anmei Shu, Jinfu Lu, Qiu Du, Yuwei Yang, Zhiyang Lv, Huiqin Xu

**Affiliations:** ^1^Hanlin College, Nanjing University of Chinese Medicine, Taizhou 225300, China; ^2^Department of Pharmacology, College of Pharmacy, Nanjing University of Chinese Medicine, Nanjing, Jiangsu 210023, China; ^3^Department of Basic Medical Science, Jiangsu Vocational College of Medicine, Yancheng, Jiangsu Province, China

## Abstract

**Background and Aims:**

Radix Rehmanniae and Corni Fructus (RC) have been widely applied to treat diabetic nephropathy (DN) for centuries. But the mechanism of how RC plays the therapeutic role against DN is unclear as yet.

**Methods:**

The information about RC was obtained from a public database. The active compounds of RC were screened by oral bioavailability (OB) and drug-likeness (DL). Gene ontology (GO) analysis was performed to realize the key targets of RC, and an active compound-potential target network was created. The therapeutic effects of RC active compounds and their key signal pathways were preliminarily probed via network pharmacology analysis and animal experiments.

**Results:**

In this study, 29 active compounds from RC and 64 key targets related to DN were collected using the network pharmacology method. The pathway enrichment analysis showed that RC regulated advanced glycosylation end product (AGE-) RAGE and IL-17 signaling pathways to treat DN. The animal experiments revealed that RC significantly improved metabolic parameters, inflammation renal structure, and function to protect the kidney against DN.

**Conclusions:**

The results revealed the relationship between multicomponents and multitargets of RC. The administratiom of RC might remit the DM-induced renal damage through the AGE-RAGE signaling pathway to improve metabolic parameters and protect renal structure and function.

## 1. Introduction

Diabetic nephropathy (DN) is a refractory chronic microvascular complication of diabetic mellitus, resulting in end-stage renal disease (ESRD) eventually [1]. The pathological hallmarks of DN are proliferating mesangial cells, thickening basement membranes, injured glomerular, and tubular cells, leading to microalbuminuria. At present, the treatment strategy for this complication is mainly focused on controlling blood glucose levels and inhibiting the renin-angiotensin system (RAS) [2]. Angiotensin-converting enzyme (ACE) inhibitors are used to reduce proteinuria levels and to restrain the progress of DN in clinics. However, some patients with DN are unsuitable for the treatment with ACE inhibitors as who meet side-effects of these medicines, such as low blood pressure and low cardiac oxygen demand. Therefore, looking for novel ways to treat DN is urgently needed.

Increasing evidence in experimental and clinical studies show that Traditional Chinese Medicine (TCM) herbs play an efficacious effect on diabetes mellitus [3–6]. For example, iridoid glycosides and polyphenols extracted from Corni Fructus (CF) can significantly improve the metabolic parameters of diabetic renal damage [7]. Besides, Radix Rehmanniae (RR) can inhibit the progression of DN by reducing blood glucose, urea nitrogen, and 5-hydroxy methyl furfural levels [8, 9]. Long clinic practice in TCM attests that coadministration of Radix Rehmanniae and Corni Fructus (RC) achieves better curative efficacy to ameliorate DN than treatment with either reagent, while the mechanism remains unclear.

As well all know, it is difficult to clarify the specific pharmacological mechanism of TCM for their multicomponent and multitarget characteristics [10]. In order to get over the defects, in the current study, we used the existing data in the database to establish a pharmacological network and to analyse the connections between active ingredients of both reagents and potential targets of the disease [11–13]. The network pharmacology is very suitable for the mechanism study of TCM. This analysis of complex network is helpful to discover the relationship among multitarget drugs, drug combinations, and their signal pathways [14].

**Figure 1:**
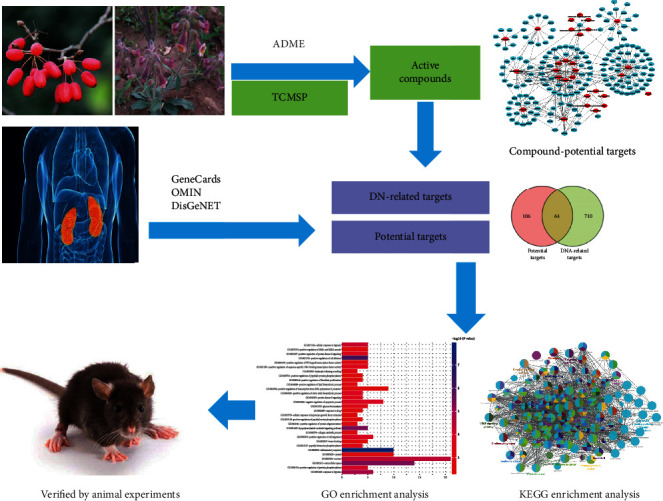
Workflow of the systematic strategies to elucidate the mechanisms of RC in the treatment of DN.

To date, the network pharmacology about the effect of RC on DN has not been reported yet [15]. Therefore, in this study, the effect of RC on DN and its protective mechanism were probed using network pharmacology analysis and relevant animal experiments (see Figure 1).

## 2. Materials and Methods

### 2.1. Data Preparation

All the chemical compositions in RC were obtained from TCM Systems Pharmacology Database and Analysis Platform (TCMSP, http://lsp.nwu.edu.cn/tcmsp.php).

### 2.2. Screening of Active Compounds

The active compounds from RR and CF were screened by a combination of drug-likeness (DL) and oral bioavailability (OB). (1) DL: As a qualitative concept used in drug design to estimate the “drug-like” degree of compounds, DL is helpful to describe the pharmacokinetics and drug properties of compounds, including solubility and chemical stability. (2) OB: Bioavailability reflects the percentage of oral dose absorbed by the gastrointestinal tract into the systemic circulation through the liver. OB value is one of the key factors to restrict the therapeutic effect of compounds after oral administration of TCM. We select DL (≥0.18) and OB (≥30%) as thresholds to screen the active compounds in RC [16]. However, some chemical constituents, not meeting the above criterion, were still classified as active ingredients for their stronger pharmacological effects and higher activities in the literature search [17, 18].

### 2.3. Prediction for Potential RC Targets

Potential targets of active compounds from RC were obtained from the TCMSP database, and those without target information were removed [19, 20]. The database provides some comprehensive, high-quality, and freely accessible protein sequence and functional information. The names of proteins were listed in UniProtKB (http://www.uniprot.org/), and the organism was taken as “Homo sapiens” before the official symbols were retrieved.

### 2.4. Collection of DN-Related Targets

DN-related targets were collected from the following databases. (1) GeneCards provides user-friendly information about annotations and predictions of human genes. (2) Online Mendelian Inheritance in Man (OMIM) profiles human genes and phenotypes. We searched for the keyword “diabetic nephropathy” and finally found the related genes [21]. (3) DisGeNET (http://www.disgenet.org/web/DisGeNET) offers information about the molecular basis of disease and predictive disease genes, etc. We searched for the keyword “diabetic nephropathy” and collected all genes [22].

### 2.5. Network Construction

We established an interaction network among herbs, active compounds, and potential targets of DN and RC. The interaction network was visualized by the Cytoscape 3.6.0 software (version3.6.0, http://www.cytoscape.org) [23].

### 2.6. Gene Ontology Analysis

We used annotation, visualization, and comprehensive discovery database (DAVID6, 6.8 edition) to carry out the functional enrichment analysis of gene ontology (GO) and to determine the biological significance of genes [24, 25]. Besides, we used ClueGO to analyze the key targets of RC. ClueGO is a Cytoscape plugin for visualizing nonredundant biological terms for gene clusters in a functional packet network, which reflects the relationship of relative gene terms [26].

### 2.7. Animal Experiment

#### 2.7.1. Animals

18 KKAy mice and 6 C57BI/6J male mice (10 weeks old, SPF grade) were purchased from Beijing Huafukang Bioscience Co., Ltd. The mice were housed in an experimental facility with an ambient temperature of 20-25°C, relative humidity of 50-60%, light/dark cycle of 12 h, and with ad libitum access to food and water. All animals used were carried out in accordance with the international guidelines for the care and use of experimental animals and approved by the ethics committee of Nanjing University of Traditional Chinese Medicine.

#### 2.7.2. Animal Modeling and Grouping

The KKAy mice were fed with a high-fat food (65.5% of common diet, 24% of lard, 10% of sugar, 0.2% of cholesterol, and 5% of egg yolk powders) for eight weeks. Then, mice with intravenous glucose level ≥ 10.0 mmol/l and 24 h urine protein ≥ 0.6 mg were used as diabetic nephropathy models. The C57bl/6J mice were fed with normal food as the normal control group.

The model mice were randomly divided into 3 groups of 6 each: model control group, aminoguanidine group, and RC group. The latter two groups were intragastrically administered with 100 mg/kg/d aminoguanidine or 3 g/kg/d RC once a day for continuous 8 weeks. All mice were given the same volume of deionized water (0.1 ml/10 g body weight) and were recorded the changes of mental state, body weights, food consumption, water intake, urine volume, and fasting blood glucose level during the experiment.

#### 2.7.3. Urinary Protein Quantitation

After administration of the drugs, the 24 h urine volume of all mice was obtained at the 0th, 4th, and 8th week, respectively. The urine was centrifuged at 3000 rpm for 15 min. The 24 h urinary protein was determined using the corresponding test kit (Nanjing Jiancheng Bioengineering Research Co., Nanjing, China) in accordance with the manufacturer's instructions.

#### 2.7.4. Sample Collection

Fasted for 12 h after the last injection, all animals were taken blood samples from the eyes. Blood glucose was determined by the blood glucose meter (Bayer HealthCare Ltd., Germany). The serum insulin levels, the AGE levels in the serum, the AGE levels in the kidney, the IL-10 levels in the serum, the IL-10 levels in the kidney, the IL-12 levels in the serum, the IL-12 levels in the kidney, the IL-17 levels in the serum, and the IL-17 levels in the kidney were measured using ELISA kits (Shanghai Enzyme-linked Biotechnology Co., Ltd., Shanghai, China) according to the protocols. The kidneys of all mice were collected and weighed, and the kidney/body ratio was calculated.

#### 2.7.5. Histological and Immunohistochemical Examination

The kidney and pancreas were fixed with 10% formalin solution and embedded in paraffin. The paraffin block was cut into 5 *μ*m thick and stained with hematoxylin and eosin (HE). After rehydration, the samples were transferred to citrate buffer (pH 7.6), heated in a microwave oven at 65°C for 20 min, then incubated with rabbit anti-RAGE antibody (Abcam, ab3611, GR316801-2) and secondary antibody. The slides were dyed with hematoxylin and analyzed with a digital camera and ImagePro Plus software.

#### 2.7.6. Western Blot

The protein was extracted from mouse kidney tissues using RIPA lysis buffer containing 1% phenylmethylsulfonyl fluoride (PMSF), protease, and phosphatase inhibitors. Protein was separated using SDS-polyacrylamide gel electrophoreses and transferred to a PVDF membrane (Millipore, USA). Afterwards, the membrane was blocked with 5% BSA and incubated with primary antibodies against RAGE, p65 NF-*κ*B, and phospho-p65 NF-*κ*B (1 : 1000 diluted), and subsequently with horseradish peroxidase-conjugated secondary antibodies. Protein bands were visualized using a chemiluminescence kit (Millipore, USA), and their intensities were quantified by the Image J software. Experiments were conducted in triplicate.

### 2.8. Statistical Analysis

All measurement data were expressed as the mean ± SEM. The statistical analyses were carried out with the SPSS 19.0 software (SPSS Inc., Chicago, IL, USA). One-way analysis of variance (ANOVA) was used to compare the differences among multiple groups. A value of *P* < 0.05 was defined as statistically significant.

## 3. Results

### 3.1. Screening of Active Compounds in RC

We found that RC contains 298 chemical components, 227 of which were derived from CF and 77 from RR through the TCMSP database. In this study, 21 active compounds from 298 chemical components were further screened according to the ADME screening criterion (DL ≥ 0.18; OB ≥ 30%). In addition, eight compounds below the screening criterion were also taken as active compounds for further analysis. For example, ursolic acid (DL = 0.75, OB = 16.77%) and catalpol (DL = 0.44, OB = 5.9%) are still considered as active components according to the literature reports, because the former can improve glomerular hypertrophy and accumulate type IV collagen (COL-IV) in STZ-induced diabetic mice [27, 28], and the latter can reduce blood glucose level as well as inhibit inflammation and oxidative stress [29, 30]. In total, 29 main active compounds were selected from RR and CF and are shown in Table 1 (detailed compounds information is presented in Supplementary Table 1).

### 3.2. Prediction of RC Target

We predicted the potential targets of RC based on their active compounds used in the system target prediction method. We forecast 294 potential targets for 29 active compounds: 246 for CF and 82 for RR. After duplicates were deleted, there were 170 targets remained. Detailed information about potential targets is presented in Supplementary Table 2.

### 3.3. Network of Active Compound-Potential Target

**Figure 2:**
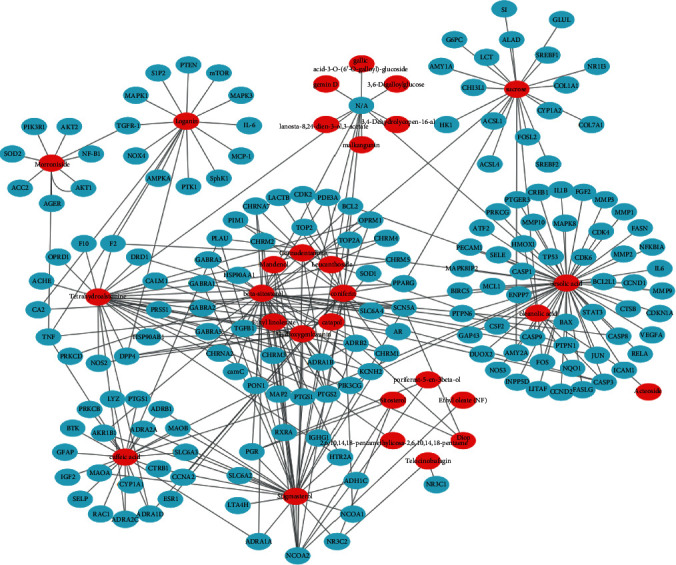
Active compound-potential target network. The C-T network was constructed by active compounds and their potential targets. The red ellipses represent the 29 active compounds, and the blue ellipses represent the 170 potential targets on which the compounds act.

The study of complex interactions between active compounds and their potential targets is helpful to understand the pharmacological mechanism of RC. We constructed a network based on active compounds and their potential targets through Cytoscape (see Figure 2). Through the analysis of the network, 29 of 199 are active compound nodes and 170 of 296 are potential target nodes. The top 10 active compounds ranked by degree are as follows: hydroxygenkwanin, morroniside, oleanolic acid, catalpol, leucanthoside, cornudentanone, sitosterol, etc. Of them, morroniside, catalpol, and oleanolic acid are the main compounds of RR and CF that are widely used in China to treat DN through antioxidation, hypoglycemic, anti-inflammatory, hypotensive, and reduction of stress [30–34]. These active compounds may exert synergistic effects to treat DN via the activation of the same targets, such as prostaglandin G/H synthase 2 (PTGS2), dipeptidyl peptidase 4 (DPP4), nuclear receptor coactivator 2 (NCOA2), nuclear factor kappa-B (NF-*κ*B), and tumor necrosis factor-alpha (TNF-*α*) [35–37].

### 3.4. Collection of DN-Related Targets

After deleting the duplicate genes, we collected 774 DN-related target genes from three disease-related databases: OMIM, GeneCards, and the DisGeNET (Supplementary Table 3). Of them, 64 DN-related targets of active compounds were found as the key targets, and they are listed in Table 2.

### 3.5. GO Functional Annotation

**Figure 3:**
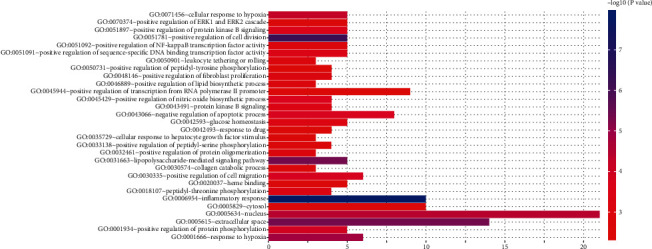
GO enrichment analysis of key targets. The number of GO entries in the functional categories of cell composition, molecular function, and biological process (FDR < 0.05).

Through GO enrichment analysis, 30 top GO entries (FDR < 0.05) were selected according to the error detection rate (FDR) as shown in Figure 3. These key targets in the network tend to reduce cell migration, proliferation, apoptosis, and differentiation by activating cell transcription factors and regulating cell inflammatory response, energy metabolism, and signal transduction.

### 3.6. Signaling Pathway Analysis

**Figure 4:**
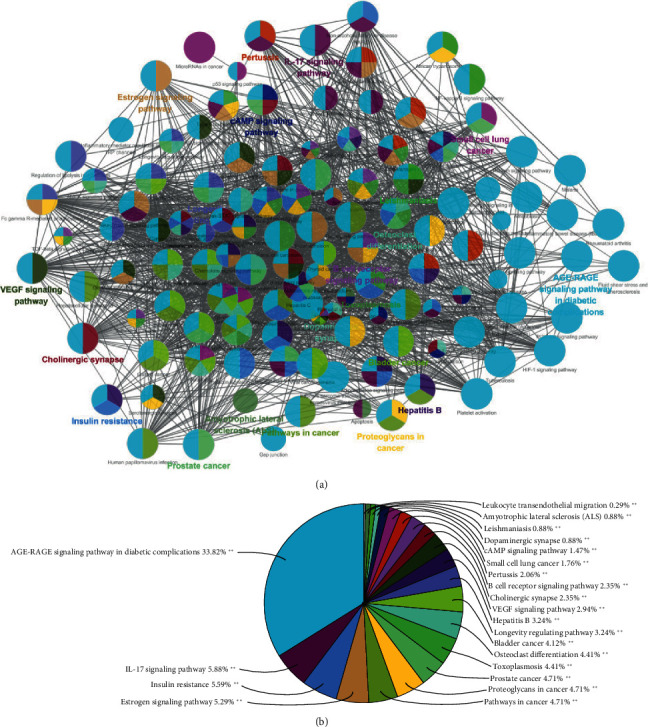
ClueGO pathway analysis. A functionally grouped network of enriched categories was generated for the key targets. The GO term was denoted as a node, and the size of the node denoted the richness of the term. Functionally related groups partially overlap. Only the most important terms in the group are marked. Representative enriched pathway (*P* < 0.05) interactions among the main RC targets.

As shown in Figure 4, the pathway enrichment analysis with ClueGO (a Cytoscape plugin) showed that key targets were mainly assigned to the AGE-RAGE and IL-17 signaling pathway. These pathways played a definite role in oxidative stress, glycolipid metabolism, inflammation, and renal fibrosis [38–40]. The typical targets of RC are regulated via a lot of pathways, such as DPP4, SOD1, ATK1, TGF-*β*1, SphK1, RAGE, MAPK3, IL-6, PTK1, NOX4, TNF-*α*, and NF-*κ*B.

### 3.7. Animal Experiment

#### 3.7.1. RC Ameliorated Diabetic Symptoms

**Figure 5:**
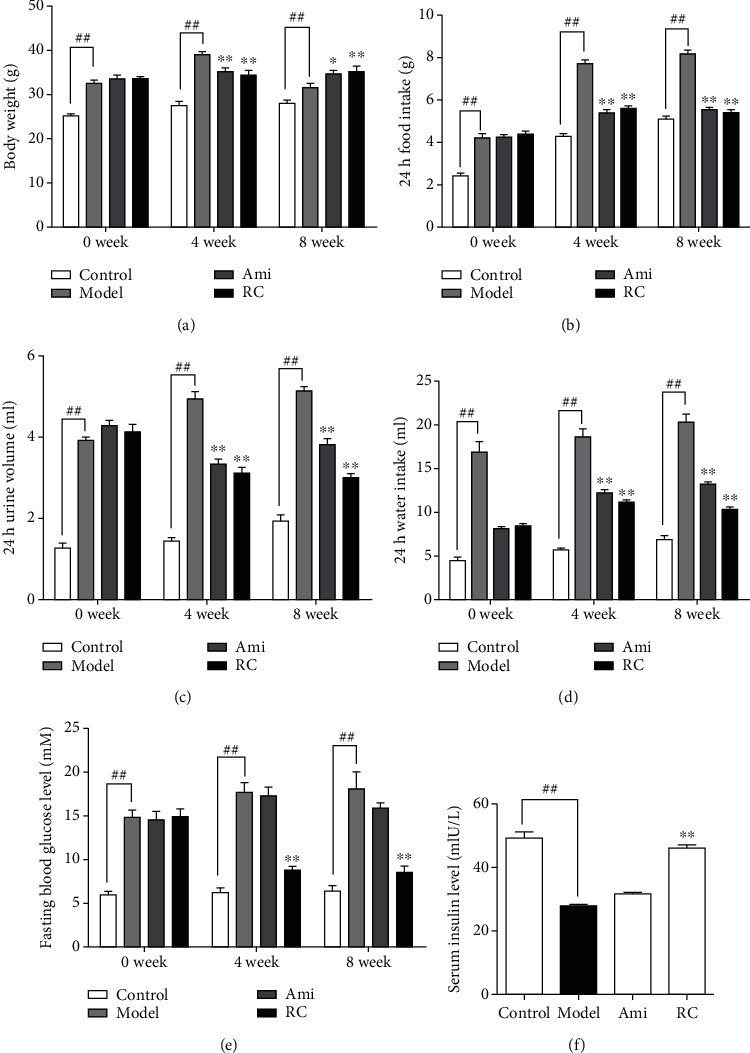
RC ameliorated the general diabetic symptoms in mice. (a) Body weights in the 0th, 4th, and 8th weeks during the experiment. (b) 24 h food intake in the 0th, 4th, and 8th weeks during the experiment. (c) 24 h urine volumes in the 0th, 4th, and 8th weeks during the experiment. (d) 24 h water intakes in the 0th, 4th, and 8th weeks during the experiment. (e) Fasting blood glucose levels in the 0th,4th, and 8th weeks during the experiment. (f) Serum insulin levels at the end of the experiment. Significance: ^##^*P* < 0.01 versus the control group, ^∗^*P* < 0.05, ^∗∗^*P* < 0.01 versus the DN group.

Body weights, food consumption, water intake, and urine volume were, respectively, recorded at the 0th, 4th, and 8th week during the animal experiment. At the 8th week, body weight, food consumption, water intake, and urine volume of DN mice increased significantly (P < 0.01) (Figures 5(a)-5(d)). Administration of aminoguanidine or RC improved these symptoms compared to the DN group (Figures 5(a)-5(d)). In addition, the fasting glucose level of DN mice increased significantly (P < 0.01), but the insulin level decreased significantly (P < 0.01) compared with the controls (Figure 5(e) and 5(d)).

#### 3.7.2. RC Improved Kidney Function of DN Mice

**Figure 6:**
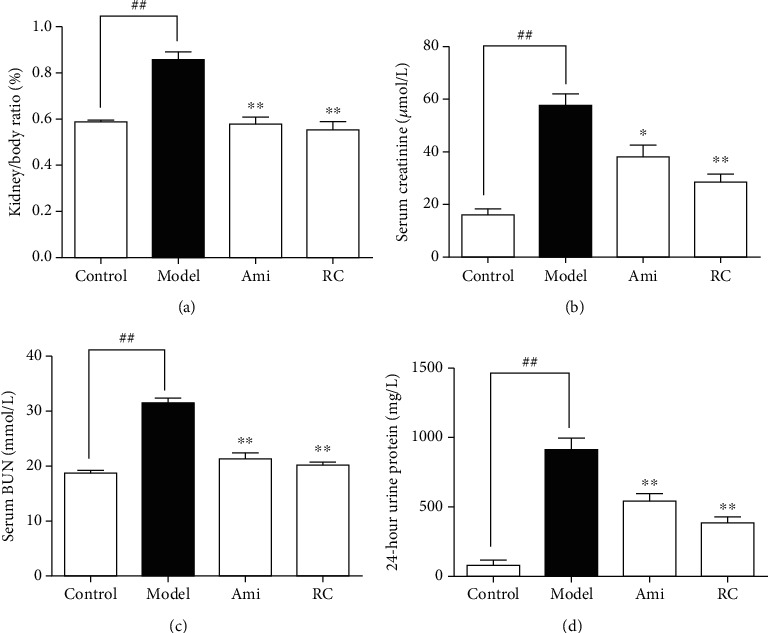
RC improved kidney function in DN mice. (a) Kidney/body weight ratios at the end of the experiments. (b) Creatinine levels in serum. (c) BUN levels in serum. (d) 24 h urine protein in the 8th week at the end of the experiment. Significance: ^##^*P* < 0.01 versus the control group, ^∗^*P* < 0.05, ^∗∗^*P* < 0.01 versus the DN group.

As shown in Figure 6, the kidney/body weight ratio of DN mice significantly decreased (P < 0.01) compared with the controls (Figure 6(a)). Treatment with aminoguanidine or RC improved the ratio compared to the DN group (Figure 6(a)). Serum creatinine (Figure 6(b)), urea nitrogen (Figure 6(c)), and 24 h urine protein levels (Figure 6(d)) in the DN group increased significantly (P < 0.01), but decreased in the RC treatment group.

#### 3.7.3. RC Improved the Inflammation of DN Mice

**Figure 7:**
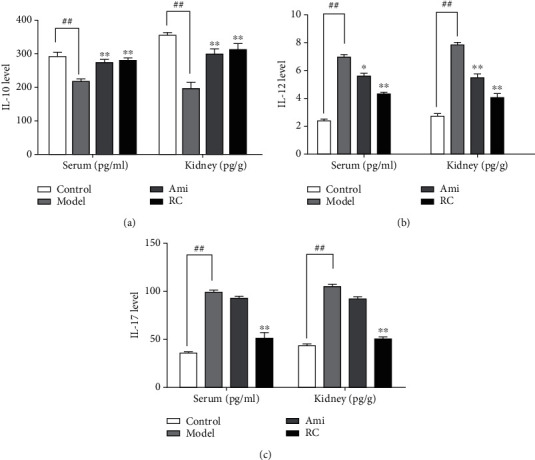
RC improved the inflammation in DN mice. (a) IL-10 levels in serum and the kidney. (b) IL-12 levels in serum and the kidney. (c) IL-17 levels in serum and the kidney. Significance: ^##^*P* < 0.01 versus the control group, ^∗^*P* < 0.05, ^∗∗^*P* < 0.01 versus the DN group.

In order to clarify the effect of inflammation on the disease, we measured the serum IL-10, IL-17, and IL-12 levels and kidney IL-10, IL-17, and IL-12 levels. The IL-10 levels decreased (P < 0.01), and IL-12 and IL-17 levels increased (P < 0.01) significantly in DN mice. Administration of aminoguanidine or RC upregulated the IL-10 levels and downregulated the IL-12 and IL-17 levels, respectively (Figure 7(a)-7(c)).

#### 3.7.4. RC Improved Kidney and Pancreas Pathohistology

**Figure 8:**
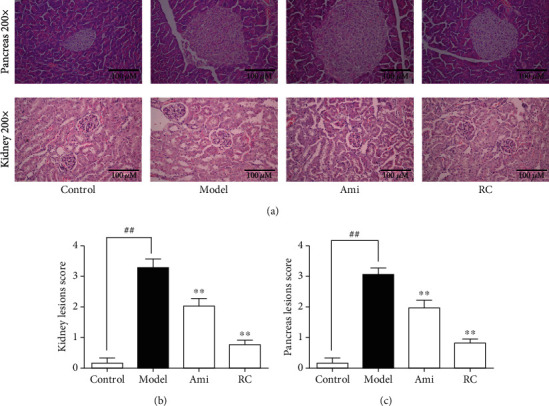
RC improved the pathohistology of the kidneys and pancreas in DN mice. (a) Images of the HE sections in the pancreas (magnification = 200x) and testes (magnifications = 200) of each group. (b) Statistical chart of the kidney lesion score. (c) Statistical chart of the pancreas lesion score. Significance: ^##^*P* < 0.01 versus the control group, ^∗^*P* < 0.05, ^∗∗^*P* < 0.01 versus the DN group.

Images of HE staining showed the increase in the mesangial matrix, cell vacuolation and degeneration, hyperplasia and hypertrophy of renal cortex, hyaline degeneration of renal tubules, and infiltration of inflammatory cells into interstitium of the kidney of DN mice, while there was little proliferation of mesangial matrix and infiltration of inflammatory cells in interstitial tissue of control mice. In addition, the pancreas of DN mice also showed vacuolar degeneration, irregular acinar cells, and disordered arrangement, while treatment with RC or aminoguanidine for 8 weeks alleviated these pathological changes (Figure 8(a)). The statistic charts for kidneys and pancreas lesion scores are shown in Figures 8(b) and 8(c).

#### 3.7.5. RC Inhibited the AGE-RAGE Pathway

**Figure 9:**
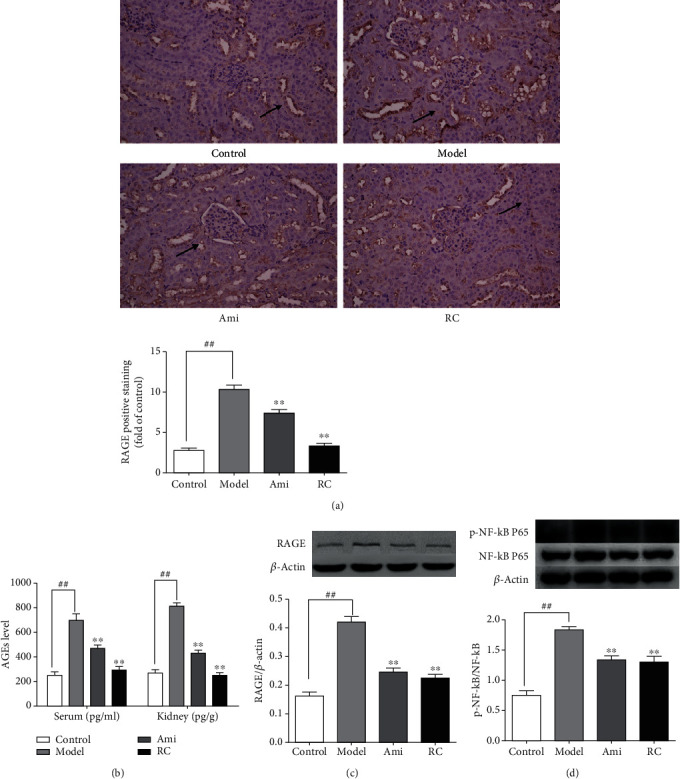
RC inhibited the AGE-RAGE pathway in the kidney of DN mice. (a) Immunohistochemistry analysis of RAGE protein expression and statistical chart of RAGE positive staining analyzed by the ImageJ software. Arrows show the protein expression. (b) AGE levels in serum and the kidney. (c) Western blot analyses of RAGE protein expression in the kidney. (d) Western blot analyses of p65 NF-*κ*B phosphorylation in the kidney and statistical chart of p-p65 NF-*κ*B/p65 NF-*κ*B ratio. *β*-Actin was used as the loading control. Bars represent the mean ± SD, *n* = 3. Significance: ^##^*P* < 0.01 versus the control group, ^∗^*P* < 0.05, ^∗∗^*P* < 0.01 versus the DN group.

In order to further explore the potential mechanism of RC, serum advanced glycation end product (AGE) levels, kidney AGE levels, RAGE receptor expression, and p65 NF-*κ*B phosphorylation in the kidney were detected. The serum AGE and kidney AGE levels in DN mice were higher (P < 0.01) than controls, but the administration of RC or aminoguanidine might downregulate the expression of AGE (Figure 9(b)). Compared with the control group, the RAGE expression increased significantly in DN mice, as evidenced by the immunohistochemistry staining and Western blot (Figures 9(a) and 9(c)). Moreover, the phosphorylation levels of p65 NF-*κ*B were elevated in DN mice (Figure 9(d)). In contrast, treatment with RC or aminoguanidine might downregulate the expression of RAGE and p-p65 NF-*κ*B (P < 0.01).

## 4. Discussion

DN is a microvascular complication of diabetes mellitus that is implicated in inflammation, adhesion molecule expression, vascular lesion, abnormal cell differentiation, migration, and proliferation. At present, there is no perfect cure for this disease. Therefore, it is urgent to find new drugs to treat this disease. We paid attention to TCM which showed great potential in the development of new drugs. Because of complicated components in TCM, it is difficult to fully clarify their effective components and related mechanism using traditional research methods. So the newly developed network pharmacology and other technologies were expected to solve the problem of multicomponent/multitarget/complex diseases of TCM. In this study, the mechanism and signal pathway of RC to alleviate DN were preliminarily explored through network pharmacological analysis and animal experiments, in order to provide direction and insight for follow-up basic and clinical research.

Through the network topology analysis, we determined the top 10 active compounds in RC according to the degree and intermediate, such as morroniside, loganin, catalpol, acteoside, ursolic acid, oleanolic acid, caffeic acid, catapol, and quercetin. Of them, the first six compounds can downregulate the NF-*κ*B signaling, while the latter three may modulate PPAR-*γ* and NF-*κ*B activities. The NF-*κ*B transcription factor is one of the abundant transcription regulators which can adjust cell inflammation and immune response. NF-*κ*B is inactive in cytoplasm as it binds to IBA. After stimulus-inducing phosphorylation of IBA, NF-*κ*B becomes active and then transfers from the cytoplasm to the nucleus to trigger gene transcription, including IL-6, TNF-*α*, IL-1 *β*, and iNOS [15].

Through the analysis of GO enrichment and KEGG pathway of key targets, it was found that RC can alleviate hyperglycemia, oxidative stress, abnormal glycolipid metabolism, inflammation, and renal fibrosis and inhibit transcription factors and cell migration to protect renal function and to delay DN progression through the regulation of AGE-RAGE and IL-17 signaling pathways.

In the AGE-RAGE signaling pathway, AGEs are produced by irreversible nonenzymatic glycosylation of reducing sugars. The accumulation of AGEs may induce endothelial cells, mesangial cells, monocytes-macrophages, and podocytes to secrete cytokines (such as FN, COL-IV, TNF-*α*), leading to extracellular matrix migration (ECM), glomerular proliferative lesion, and renal tubular dysfunction. Besides, AGEs can bind to RAGE receptors on the cell surface to activate intracellular signaling pathways, such as AGEs-RAGE, NF-*κ*B, and AGEs-RAGE-TGF*β*1, leading to release various chemokines and growth factors, proliferate glomerular mesangial cells, and abduct podocyte apoptosis; these eventually aggravate DN [41, 42]. The serum and kidney AGE content and the RAGE expression in the DN group were higher than those in the controls and were reduced by treatment with RC. Of the top 10 active compounds in RC, morroniside and loganin can downregulate the mRNA and protein expression of RAGE and upregulation of AGE levels to play a part in the protective effect against DN. In addition, catalpol suppresses the AGE-mediated inflammation by decreasing the ROS level and NF-*κ*B activity.

In the IL-17 signaling pathway, Th17 is a new type of proinflammatory CD4^+^ T-effector cells, which are different from Th1 and Th2 cell lines [43]. The increase in Th17 cytokines (such as IL-17A) may promote the secretion of proinflammatory factors and infiltration of macrophages and aggravate the diabetes-induced renal damage in DN [44].

In the current experiment, the administration of RC and aminoguanidine reduced IL-12, blood sugar, serum creatinine, urine protein, and nitrogen levels, but increased the IL-10 secretion, and improved the symptoms of proteinuria in DN mice, suggesting that RC and aminoguanidine might improve the renal function of the DN models. Between both reagents, the effects of RC were better than aminoguanidine. The HE staining of kidneys and pancreas in DN mice showed the increase in mesangial matrix, hyperplasia and hypertrophy of renal cortex, vacuolation and degeneration of cells, hyaline degeneration of renal tubules, and infiltration of inflammatory cells in the interstitium. Besides, the pancreas of DN mice also showed vacuolar degeneration, irregular acinar cells, and disordered arrangement. The treatment with RC or aminoguanidine might alleviate the above pathological changes. Further investigations are still needed to clarify the detailed mechanism for RC-inducing alleviation of renal damage in DN subjects.

## 5. Conclusions

The characteristics of TCM are with its multicomponent, multitarget, and multichannel. In this study, the information about RC was obtained from a lot of public databases. The network pharmacology method was used to collect 29 active compounds from RC and 64 key targets related to DN. The pathway enrichment analysis showed that RC could significantly improve the metabolic parameters, renal structure, and function mainly via AGE-RAGE and IL-17 signaling pathway to treat DN. The animal experiments revealed that RC could significantly improve metabolic parameters, inflammation renal structure, and function to protect the kidney against DN.

## Figures and Tables

**Table 1:** Active compounds of RR and CF.

No.	Molecule ID	Molecule name	OB (%)	DL	Herb
1	MOL001494	Mandenol	42	0.19	CF
2	MOL001495	Ethyl linolenate	46.1	0.2	CF
3	MOL001771	Poriferast-5-en-3beta-ol	36.91	0.75	CF
4	MOL002879	Diop	43.59	0.39	CF
5	MOL002883	Ethyl oleate (NF)	32.4	0.19	CF
6	MOL003137	Leucanthoside	32.12	0.78	CF
7	MOL000358	Beta-sitosterol	36.91	0.75	CF
8	MOL000358	Sitosterol	36.91	0.75	CF, RR
9	MOL000449	Stigmasterol	43.83	0.76	CF, RR
10	MOL005360	Malkangunin	57.71	0.63	CF
11	MOL005481	2,6,10,14,18-Pentamethylicosa-2,6,10,14,18-pentaene	33.40	0.24	CF
12	MOL005486	3,4-Dehydrolycopen-16-al	46.64	0.49	CF
13	MOL005489	3,6-Digalloylglucose	31.42	0.66	CF
14	MOL005503	Cornudentanone	39.66	0.33	CF
15	MOL005530	Hydroxygenkwanin	36.47	0.27	CF
16	MOL005531	Telocinobufagin	69.99	0.79	CF
17	MOL008457	Tetrahydroalstonine	32.42	0.81	CF
18	MOL000554	Gallic acid-3-O-(6′-O-galloyl)-glucoside	30.25	0.67	CF
19	MOL005552	Gemin D	68.83	0.56	CF
20	MOL005557	Lanosta-8,24-dien-3-ol,3-acetate	44.30	0.82	CF
21	MOL005499	Morroniside	3.98	0.50	CF
22	MOL001680	Loganin	5.90	0.44	CF
23	MOL000511	Ursolic acid	16.77	0.75	CF
24	MOL000414	Caffeic acid	54.97	0.05	CF
25	MOL000263	Oleanolic acid	29.02	0.76	CF
26	MOL002819	Catalpol	5.07	0.44	RR
27	MOL003333	Acteoside	2.94	0.62	RR
28	MOL000519	Coniferin	31.11	0.32	RR
29	MOL000842	Sucrose	7.17	0.23	RR

Abbreviations: CF: Corni Fructus; RR: Radix Rehmanniae; OB: oral bioavailability; DL: drug-likeness.

**Table 2:** Sixty-four DN-related targets of active compounds.

Uniprot IDs	Protein name	Gene name
P22303	Acetylcholinesterase	ACHE
P08588	Beta-1 adrenergic receptor	ADRB1
Q15109	Advanced glycosylation end product-specific receptor	AGER
P15121	Aldo-keto reductase family 1 member B1	AKR1B1
P31749	RAC-alpha serine/threonine-protein kinase	AKT1
P31751	RAC-beta serine/threonine-protein kinase	AKT2
P10415	Apoptosis regulator Bcl-2	BCL2
P42574	Caspase-3	CASP3
P24385	G1/S-specific cyclin-D1	CCND1
P02452	Collagen alpha-1(I) chain	COL1A1
P16220	Cyclic AMP-responsive element-binding protein 1	CREB1
P27487	Dipeptidyl peptidase 4	DPP4
P21728	D(1A) dopamine receptor	DRD1
P03372	Estrogen receptor	ESR1
P00734	Prothrombin	F2
P48023	Tumor necrosis factor ligand superfamily member 6	FASLG
P09038	Fibroblast growth factor 2	FGF2
P09601	Heme oxygenase 1	HMOX1
P28223	5-Hydroxytryptamine receptor 2A	HTR2A
P05362	Intercellular adhesion molecule 1	ICAM1
P01344	Insulin-like growth factor II	IGF2
P01584	Interleukin-1 beta	IL1B
P05231	Interleukin-6	IL6
P05412	Transcription factor AP-1	JUN
P61626	Lysozyme C	LYZ
P28482	Mitogen-activated protein kinase 1	MAPK1
P27361	Mitogen-activated protein kinase 3	MAPK3
P45983	Mitogen-activated protein kinase 8	MAPK8
P03956	Interstitial collagenase	MMP1
P09238	Stromelysin-2	MMP10
P08253	72 kDa type IV collagenase	MMP2
P08254	Stromelysin-1	MMP3
P14780	Matrix metalloproteinase-9	MMP9
P42345	Serine/threonine-protein kinase mTOR	mTOR
P35228	Nitric oxide synthase type II	NOS2
P29474	Nitric oxide synthase type III	NOS3
Q9NPH5	NADPH oxidase 4	NOX4
P15559	NAD(P)H dehydrogenase [quinone] 1	NQO1
P04150	GR (nuclear receptor subfamily 3 group C member 1)	NR3C1
P08235	Nuclear receptor subfamily 3, group C, member 2 variant 1	NR3C2
P48736	Phosphatidylinositol 4,5-bisphosphate 3-kinase catalytic subunit gamma isoform	PIK3CG
P27986	Phosphatidylinositol 3-kinase regulatory subunit alpha	PIK3R1
P00749	Urokinase-type plasminogen activator	PLAU
P27169	Serum paraoxonase/arylesterase 1	PON1
P37231	Peroxisome proliferator-activated receptor gamma	PPARG
P05771	Protein kinase C beta type	PRKCB
Q05655	Protein kinase C delta type	PRKCD
P07477	Trypsin-1	PRSS1
P60484	Phosphatidylinositol 3,4,5-trisphosphate 3-phosphatase and dual-specificity protein phosphatase PTEN	PTEN
P23219	Prostaglandin G/H synthase 1	PTGS1
P35354	Prostaglandin G/H synthase 2	PTGS2
P18031	Tyrosine-protein phosphatase nonreceptor type 1	PTPN1
P29350	Tyrosine-protein phosphatase nonreceptor type 6	PTPN6
Q04206	Transcription factor p65	RELA
P16581	E-selectin	SELE
P16109	P-selectin	SELP
P00441	Superoxide dismutase (Cu-Zn)	SOD1
P04179	Superoxide dismutase (Mn)	SOD2
Q9NYA1	Sphingosine kinase 1	SphK1
P40763	Signal transducer and activator of transcription 3	STAT3
P01137	Transforming growth factor beta-1 proprotein	TGFB1
P01375	Tumor necrosis factor	TNF
P04637	Cellular tumor antigen p53	TP53
P15692	Vascular endothelial growth factor A	VEGFA

## Data Availability

The data used to support the findings of this study are available from the corresponding author upon request.

## Supplementary Materials

Supplementary Materials Table S1: 298 compounds information. Table S2: 170 potential targets of RC. Table S3: DN-related targets.

## References

[1] Sun Y., Su Y., Li J., Wang L. F. (2013). Recent advances in understanding the biochemical and molecular mechanism of diabetic nephropathy. *Molecular cell biology research communications*.

[2] Gandhi S., Srinivasan B. P., Akarte A. S. (2012). Effective blockade of RAAS by combination of aliskiren and olmesartan improves glucose homeostasis, glomerular filtration rate along with renal variables in streptozotocin induced diabetic rats. *European Journal of Pharmaceutical Sciences Official Journal of the European Federation for Pharmaceutical Sciences.*.

[3] Tzeng T. F., Liou S. S., Liu I. (2013). The selected traditional Chinese medicinal formulas for treating diabetic nephropathy: perspective of modern science. *Journal of Traditional and Complementary Medicine*.

[4] Shi X., Lu X. G., Zhan L. B. (2011). The effects of the Chinese medicine ZiBu PiYin recipe on the hippocampus in a rat model of diabetes-associated cognitive decline: a proteomic analysis. *Diabetologia*.

[5] Wen X., Zeng Y., Liu L. (2012). Zhenqing recipe alleviates diabetic nephropathy in experimental type 2 diabetic rats through suppression of SREBP-1c. *Journal of Ethnopharmacology*.

[6] Tong X. L., Dong L., Chen L., Zhen Z. (2012). Treatment of diabetes using traditional Chinese medicine: past, present and future. *The American Journal of Chinese Medicine*.

[7] Yamabe N., Kang K. S., Matsuo Y., Tanaka T., Yokozawa T. (2007). Identification of antidiabetic effect of iridoid glycosides and low molecular weight polyphenol fractions of Corni fructus, a constituent of Hachimi-jio-gan, in streptozotocin-induced diabetic rats. *Biological & Pharmaceutical Bulletin*.

[8] Wang Z., Wang J., Chan P. (2013). Treating type 2 diabetes mellitus with traditional Chinese and Indian medicinal herbs. *Evidence-Based Complementray and Alternative Medicine*.

[9] Takako Y., Hyun Y. K., Noriko Y. (2014). Amelioration of diabetic nephropathy by dried Rehmanniae Radix (Di Huang) extract. *American Journal of Chinese Medicine*.

[10] Hopkins A. L. (2008). Network pharmacology: the next paradigm in drug discovery. *Nature Chemical Biology*.

[11] Hopkins A. L. (2007). Network pharmacology. *Nature Biotechnology*.

[12] Ru J., Li P., Wang J. (2014). TCMSP: a database of systems pharmacology for drug discovery from herbal medicines. *Journal of Cheminformatics*.

[13] Liu J., Pei M., Zheng C. (2013). A systems-pharmacology analysis of herbal medicines used in health improvement treatment: predicting potential new drugs and targets. *Evidence-Based Complementary and Alternative Medicine*.

[14] Keith C. T., Borisy A. A., Stockwell B. R. (2005). Multicomponent therapeutics for networked systems. *Nature Reviews. Drug Discovery*.

[15] Li S., Zhang B., Zhang N. (2011). Network target for screening synergistic drug combinations with application to traditional Chinese medicine. *Bmc Systems Biology*.

[16] Plewczynski D., Philips A., Grotthuss M. V., Rychlewski L., Ginalski K. (2014). HarmonyDOCK: the structural analysis of poses in protein-ligand docking. *Journal of Computational Biology A Journal of Computational Molecular Cell Biology*.

[17] Yang H., Zhang W., Huang C. (2014). A novel systems pharmacology model for herbal medicine injection: a case using reduning injection. *BMC complementary medicine and therapies*.

[18] Yue S. J., Liu J., Feng W. W. (2017). System pharmacology-based dissection of the synergistic mechanism of Huangqi and Huanglian for diabetes mellitus. *Frontiers in Pharmacology*.

[19] Yao Y., Zhang X., Wang Z. (2013). Deciphering the combination principles of Traditional Chinese Medicine from a systems pharmacology perspective based on Ma-huang decoction. *Journal of Ethnopharmacology*.

[20] Yu H., Chen J., Xu X. (2012). A systematic prediction of multiple drug-target interactions from chemical, genomic, and pharmacological data. *PLoS One*.

[21] Hamosh A., Scott A. F., Amberger J. (2004). Online Mendelian Inheritance in Man (OMIM), a knowledgebase of human genes and genetic disorders. *Nucleic Acids Research*.

[22] Pinero J., Queralt-Rosinach N., Bravo À. (2015). DisGeNET: a discovery platform for the dynamical exploration of human diseases and their genes. *Database*.

[23] Shannon P., Markiel A., Ozier O. (2003). Cytoscape: a software environment for integrated models of biomolecular interaction networks. *Genome Research*.

[24] Sherlock G. (2000). Gene Ontology: tool for the unification of biology. *Canadian Institute of Food Science & Technology Journal*.

[25] Huang D. W., Sherman B. T., Lempicki R. A. (2009). Systematic and integrative analysis of large gene lists using DAVID bioinformatics resources. *Nature Protocols*.

[26] Bindea G., Mlecnik B., Hackl H. (2009). ClueGO: a Cytoscape plug-in to decipher functionally grouped gene ontology and pathway annotation networks. *Bioinformatics*.

[27] Zhou Y., Li J. S., Zhang X. (2010). Ursolic acid inhibits early lesions of diabetic nephropathy. *International Journal of Molecular Medicine*.

[28] Zhang M. F., Shen Y. Q. (2016). *Research Advances in Pharmacologic Effects of Ursolic Acid in Treating Antidiabetic Complications, Anti-Infection Pharmacy*.

[29] Zhang H., Jia R., Wang F. (2018). Catalpol protects mice against lipopolysaccharide/D-galactosamine-induced acute liver injury through inhibiting inflammatory and oxidative response. *Oncotarget*.

[30] Dong Z., Chen C. X. (2013). Effect of catalpol on diabetic nephropathy in rats. *Phytomedicine*.

[31] Lee E., Kim H. M., Kang J. S. (2016). Oleanolic acid and N-acetylcysteine ameliorate diabetic nephropathy through reduction of oxidative stress and endoplasmic reticulum stress in a type 2 diabetic rat model, nephrology, dialysis, transplantation: official publication of the European Dialysis and Transplant Association - European Renal Association. *Nephrology Dialysis Transplantation*.

[32] Yokozawa T., Yamabe N., Kim H. Y. (2008). Protective effects of morroniside isolated from Corni Fructus against renal damage in streptozotocin-induced diabetic rats. *Biological & Pharmaceutical Bulletin*.

[33] Jiang W. L., Zhang S. P., Hou J., Zhu H. B. (2012). Effect of loganin on experimental diabetic nephropathy. *Phytomedicine : international journal of phytotherapy and phytopharmacology*.

[34] Liu K., Xu H., Lv G. (2015). Loganin attenuates diabetic nephropathy in C57BL/6J mice with diabetes induced by streptozotocin and fed with diets containing high level of advanced glycation end products. *Life Sciences*.

[35] Choi H. J., Jang H. J., Chung T. W. (2013). Catalpol suppresses advanced glycation end-products-induced inflammatory responses through inhibition of reactive oxygen species in human monocytic THP-1 cells. *Fitoterapia*.

[36] Feng S., Gan L., Yang C. S. (2018). Effects of stigmasterol and *β*-sitosterol on nonalcoholic fatty liver disease in a mouse model: a Lipidomic analysis. *Journal of Agricultural and Food Chemistry*.

[37] Alter M. L., Ott I. M., Von W. K. (2014). Linagliptin: an update of its use in patients with type 2 diabetes mellitus. *Drugs*.

[38] Tan A. J., Forbes J. M., Cooper M. E. (2007). AGE, RAGE, and ROS in diabetic nephropathy. *Seminars in Nephrology*.

[39] Sakurai S., Yonekura H., Yamamoto Y. (2003). The AGE-RAGE system and diabetic nephropathy. *Journal of the American Society of Nephrology*.

[40] Yamamoto Y., Kato I., Doi T. (2002). The role of AGE-RAGE system in the development of diabetic nephropathy in vivo. *International Congress Series*.

[41] Kumar Pasupulati A., Chitra P. S., Reddy G. B. (2016). Advanced glycation end products mediated cellular and molecular events in the pathology of diabetic nephropathy. *Biomolecular Concepts*.

[42] Ojima A., Matsui T., Nishino Y. (2015). Empagliflozin, an inhibitor of sodium-glucose cotransporter 2 exerts anti-inflammatory and antifibrotic effects on experimental diabetic nephropathy partly by suppressing AGEs-receptor axis. *Hormone and Metabolic Research*.

[43] Ururahy M. A. G., de Souza K. S. C., Oliveira Y. M. d. C. (2015). Association of polymorphisms inIL6gene promoter region with type 1 diabetes and increased albumin-to-creatinine ratio. *Diabetes/Metabolism Research and Reviews*.

[44] Wang B., Yao K., Wise A. F. (2017). MicroRNA-378 reduces mesangial hypertrophy and kidney tubular fibrosis via MAPK signaling. *Clinical Science*.

